# A Study on the Immunohistochemical Expressions of Leptin and Leptin Receptor in Clear Cell Renal Cell Carcinoma

**DOI:** 10.1155/2020/3682086

**Published:** 2020-08-04

**Authors:** Komathi Perumal, Kein Seong Mun, Ning Yi Yap, Azad Hassan Abdul Razack, Glenda Carolyn Gobe, Teng Aik Ong, Shanggar Kuppusamy, Retnagowri Rajandram

**Affiliations:** ^1^Department of Surgery, Faculty of Medicine, University of Malaya, Kuala Lumpur, Malaysia; ^2^Department of Pathology, Faculty of Medicine, University of Malaya, Kuala Lumpur, Malaysia; ^3^Centre of Kidney Disease Research and School of Biomedical Sciences, University of Queensland, Australia

## Abstract

**Background:**

The mechanisms that link obesity and cancer development are not well-defined. Investigation of leptin and leptin receptor expressions may help define some of the mechanisms. These proteins are known for associating with the immune response, angiogenesis and, signalling pathways such as JAK2/STAT3, PI3K, and AKT pathways. Tissue proteins can be easily detected with immunohistochemistry (IHC), a technique widely used both in diagnostic and research laboratories. The identification of altered levels of leptin and leptin receptor proteins in tumour tissues may lead to targeted treatment for cancer.

**Objective:**

The objective of this study was to use IHC to compare leptin and leptin receptor expressions in clear cell renal cell carcinomas (ccRCC) in non-obese and obese patients to determine the association between these proteins with the clinicopathological features and prognosis of ccRCC. *Patients and Methods*. The study involved 60 patients who underwent nephrectomy of which 34 were obese, as assessed using body mass index (BMI). Nephrectomy samples provided tissues of ccRCC and adjacent non-cancerous kidney. The intensity and localization of leptin and leptin receptor protein expressions were evaluated using IHC and correlated with clinicopathological features and clinical outcomes. Aperio ImageScope morphometry and digital pathology were applied to assess the IHC results. The chi-square test was used to determine if there was any significant association between the proteins and the clinicopathological features. The Kaplan-Meier test was used to determine the overall survival, disease-free survival, and recurrence-free survival. A value of *p* < 0.05 was considered significant.

**Results:**

There was neither significant difference in the overall cellular and nuclear expressions of leptin and leptin receptor between non-cancerous kidney and ccRCC tissues nor in non-obese and obese individuals with ccRCC.

**Conclusion:**

In this present study, it was revealed that leptin and leptin receptor were not associated with tumour characteristics and progression of ccRCC patients. Interestingly, nuclear expression of leptin was significantly associated with overall survival. However, the significance of these proteins as biomarkers in other RCC histotypes is still unclear.

## 1. Introduction

Renal cell carcinoma (RCC) constitutes 90% of all renal malignancies, and there is an increasing trend in the incidence of RCC worldwide. Clear cell RCC (ccRCC) is the most common subtype of RCC, comprising approximately 80% of all RCC [1]. The most commonly known risk factors for RCC include age, gender, smoking, hypertension, and obesity [2]. Other kidney diseases such as Von Hippel-Lindau/VHL an autosomal dominant hereditary disorder and end-stage renal failure also contribute to RCC [3, 4]. The incidence of obesity has also increased worldwide. In Malaysia, a 10-year survey showed that there was a significant increase in the overweight and obese population due to an inactive lifestyle [5]. Considering the obesity statistics, the overall hypothesis of the present study is that there is a causative link between obesity and RCC development.

**Figure 1:**
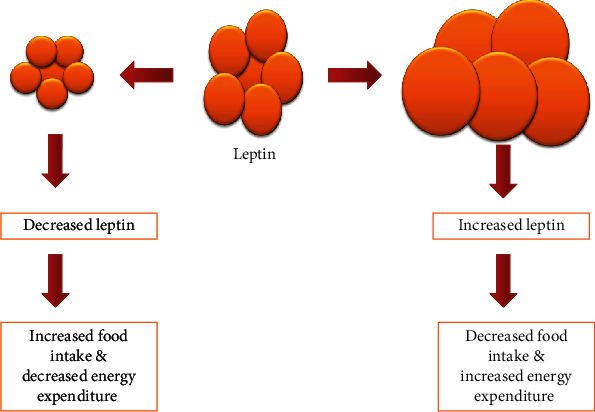
Homeostasis of leptin. Description on the role of leptin in energy expenditure and food intake.

Leptin is one of the adipokines produced from adipose tissue. Its expression is of research interest due to its role in obesity and cancer [6]. Leptin maintains the homeostasis of the human body by lowering the caloric intake and increasing energy expenditure as illustrated in Figure 1 [7]. In the process of leptin homeostasis, other pathways are activated, namely, the JAK2/STAT3, PI3K, and AKT pathways. These pathways are responsible for increasing the expression of antiapoptotic protein (X-linked inhibitor of apoptosis protein/XIAP), increasing systematic inflammation (tumor necrosis factor-*α*/TNF-*α*; interleukin-6/IL-6), and promoting angiogenic factors (vascular endothelial growth factor/VEGF and hypoxia-inducible factor-1*α*/HIF-1*α*) which foster cancer cell survival, proliferation, and migration. These factors have shown a significant association with leptin in breast cancer [8, 9] and colorectal cancer [10, 11]. Reports indicated that the presence of high serum leptin leads to the progression of cancer. To date, serum leptin has been the focus of studies investigating its contribution in the development of RCC [12, 13], with some studies indicating that high serum leptin is associated with the development and progression of RCC [14], while other studies showed no connection between leptin and the development and advancement of RCC [15].

Leptin receptor is a protein that is found extensively in the hypothalamus of the human brain and also in other peripheral tissues such as the liver, gall bladder, and colon [16]. Several studies have been carried out to correlate enhanced leptin receptor levels with carcinogenesis and cancer progression [17–19]. A study by Ishikawa and colleagues confirmed that upregulated leptin receptor expression promotes carcinogenesis and metastasis of breast cancer [9, 20]. However, there was no study on leptin receptor in association with ccRCC.

Studies on the prognostic value of leptin and leptin receptor in ccRCC are very limited especially studies using immunohistochemistry (IHC). In recent years, the number of molecular-based assays has increased but histopathology remains the gold standard for most diagnostic and therapeutic decisions. IHC is a globally available tool that complements histopathological analysis by detecting protein expression [21]. In the present study, we aim to measure the expressions of leptin and leptin receptor in ccRCC and adjacent non-cancerous tissue to determine the prognostic significance of these two proteins using IHC methods [22].

## 2. Methodology

### 2.1. Subject Recruitment and Study Samples

All samples were from patients undergoing radical nephrectomy at University Malaya Medical Centre (UMMC) between the years 2011 and 2018. The study was approved by the Ethics Committee (ID no.: 20166-2611) of the UMMC. Informed consent was obtained from all subjects whose samples were used in this study as per protocol.

### 2.2. Immunohistochemistry

Formalin-fixed paraffin-embedded (FFPE) samples were sectioned onto Superfrost Plus slides at 4 *μ*m, dewaxed in xylene, and rehydrated in several changes of alcohol to Tris-buffered saline with Tween 20 (TBST). Antigen retrieval was performed in sodium citrate buffer (pH 6 at 95°C for 20 minutes), followed by washing with TBST and blocking for 15 minutes with 3% hydrogen peroxide. Primary antibodies were obtained from Santa Cruz Biotechnology (Dallas, USA). Leptin (anti-rabbit; #sc-842; 1 : 200) and leptin receptor (anti-rabbit; #sc-8325; 1 : 100) was added to sections to bind with the protein of interest. They were placed in a humidity chamber overnight at 4°C, followed by 45 minutes of incubation with polymer as secondary antibody at room temperature. After washing with TBST, 3,3′-diaminobenzidine (DAB) staining was performed, followed by counterstaining with hematoxylin, dehydration, and mounting in Depex. All the slides were scanned using Aperio Image Scanning by Leica Biosystem at 40x magnification. The immunopositivity score was reported manually and also as a ratio of positive pixels [23]. The cut-off points were determined by using the formula below:

(1) $$\begin{array}{c} \text{Protein expression ratio}=\frac{\text{Positive pixels of ccRCC tissue}}{\text{Positive pixels of adjacent tissue}} \end{array}$$

A cut-off point of ≤0.75 was considered low expression, 0.76–1.24 was considered showing no differential expression, and ≥1.25 was considered showing increased differential expression for either proteins [24].

### 2.3. Statistical Analysis

All data were presented as a percentage, and SPSS20.0 software was used for statistical analysis. Quantitative data were analyzed by the Chi-square test, and the Kaplan-Meier test was carried out for survival analysis. *p* < 0.05 was considered statistically significant [23].

## 3. Results

Tissue samples for this study were obtained from patients who have undergone nephrectomy for RCC with subsequent histopathological confirmation of ccRCC. Based on the World Health Organisation (WHO) criteria, BMI of 18.5-24.9 is considered normal and BMI of 30–39.9 is considered obese [25]. Since the study was focused only on non-obese versus obese patients, underweight subjects (BMI of <18) and overweight subjects (BMI of 25–29.9) were excluded. The pathological diagnosis of ccRCC was confirmed by a pathologist in all samples used in this study. The clinical information for all patients was retrieved from the medical records of the UMMC.

The samples included Stage I (*n* = 23), Stage II (*n* = 14), Stage III (*n* = 12), and Stage IV (*n* = 11) ccRCC according to clinical stage. Among the patients included in this cohort, 26 have normal BMI and 34 were obese, based on WHO criteria. The demographics of the recruited ccRCC patients are shown in Table 1. In Table 2, demographic data with leptin and leptin receptor expressions are shown.

**Figure 2:**
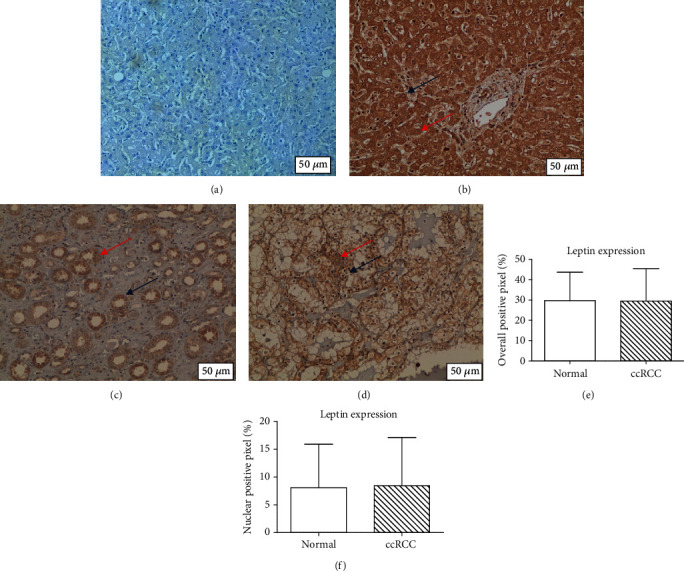
Leptin overall and nuclear immunohistochemistry. (a) Negative control. (b) Positive liver control (cytoplasm positivity indicated by the red arrow; the nucleus remains unstained as indicated by the blue arrow). (c) Adjacent normal kidney (cytoplasm positivity indicated by the red arrow; the nucleus remains unstained as indicated by the blue arrow). (d) ccRCC (focal cytoplasmic positivity indicated by the red arrow; some of the ccRCC nucleus stained positive as indicated by the blue arrow). (e) Overall positive pixel. (f) Nuclear positive pixel. There was no differential overall and nuclear expression intensity of leptin in ccRCC compared with paired normal kidney.

**Figure 3:**
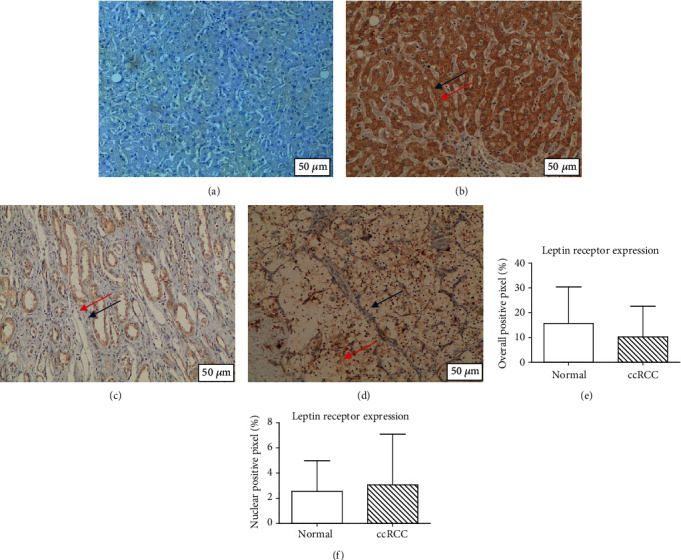
Leptin receptor overall and nuclear immunohistochemistry. (a) Negative control. (b) Positive liver control (cytoplasm positivity indicated by the red arrow; the nucleus remains unstained as indicated by the blue arrow). (c) Adjacent normal kidney (cytoplasm positivity indicated by the red arrow; the nucleus remains unstained as indicated by the blue arrow). (d) ccRCC (focal weak cytoplasmic positivity indicated by the red arrow, some of the ccRCC nucleus stained positive as indicated by the blue arrow). (e) Overall positive pixel. (f) Nuclear positive pixel. There was no differential overall and nuclear expression intensity of the leptin receptor in ccRCC compared with paired normal kidney.

Quantitative analysis of the expression intensity revealed that leptin overall positivity (cytoplasm and membrane) was not significantly different in adjacent non-cancerous kidney compared to ccRCC tissue (*p* > 0.05). There was also no difference for nuclear positivity in adjacent noncancer kidney compared to ccRCC (*p* > 0.05) as shown in Figure 2. Quantitative analysis of the expression intensity revealed there was no significant difference in adjacent non-cancerous kidney compared to ccRCC tissue for leptin receptor overall positivity and in adjacent non-cancerous kidney compared to ccRCC tissue for leptin receptor nuclear positivity (*p* > 0.05). These results are shown in Figure 3.

**Figure 4:**
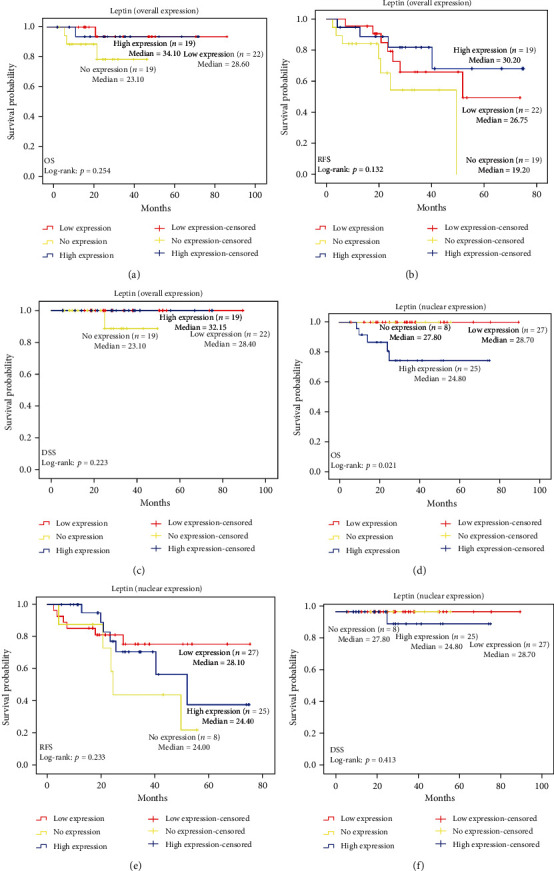
Survival rate of ccRCC associated with leptin overall and nuclear expression. (a) OS associated with leptin overall expression, (b) RFS associated with leptin overall expression, and (c) DSS associated with leptin overall expression, (d) OS associated with leptin nuclear expression, (e) RFS associated with leptin nuclear expression, and (f) DSS associated with leptin nuclear expression.

**Figure 5:**
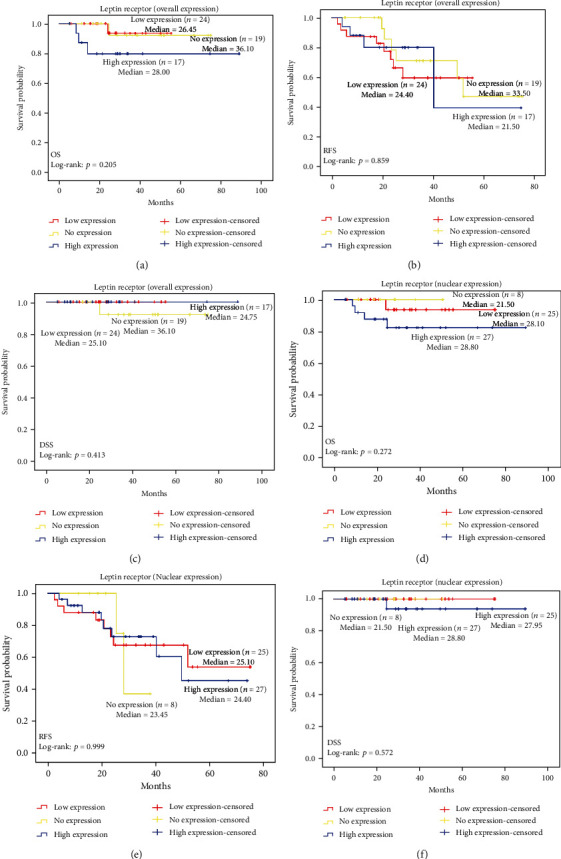
Survival rate of ccRCC associated with leptin receptor overall and nuclear expression. (a) OS associated with leptin receptor overall expression, (b) RFS associated with leptin receptor overall expression, and (c) DSS associated with leptin receptor overall expression. (d) OS associated with leptin receptor nuclear expression, (e) RFS associated with leptin receptor nuclear expression, and (f) DSS associated with leptin receptor nuclear expression.

The survival rate of recruited subjects was determined by using the Kaplan-Meier analysis based on overall survival (OS), recurrent-free survival (RFS), and disease-specific survival (DSS) (Figure 4). A cut-off point of ≥1.25 was used as a high expression in leptin overall expression and nuclear expression for OS, RFS, and DSS. Patients grouped as leptin high overall expression and nuclear expressions were not significantly associated with worse survival prognosis except for OS which was statistically significant with a *p* value of 0.021 (Figure 4(d)). A cut-off point of ≥1.25 was used as a high expression in leptin receptor overall expression and nuclear expression for OS, RFS, and DSS. Patients grouped as leptin receptor high overall expression and nuclear expressions were not significantly associated with worse survival prognosis. These data are shown in Figure 5.

## 4. Discussion

Leptin and leptin receptor proteins are primarily secreted by adipose tissue, with the major purpose being to balance the energy expenditure and caloric intake of the human body. The activation of these proteins can also activate pathways such as STAT3/JAK2, P13K, and AKT that promote the proliferation, progression, and survival of cancerous cells [26]. Examples of molecular changes include the antiapoptotic protein XIAP whose expression is elevated in many cancer types and participate in the release of proapoptotic proteins [27]; inflammation mediators such as TNF-*α*, IL-6, transforming growth factor-*β*, and IL-10 have been shown to participate in both the initiation and progression of cancer [28]; and the VEGF signaling pathway plays pivotal roles in regulating tumour angiogenesis [29]. Several studies have reported that large amounts of adipose tissue in the body can lead to cancer progression; for example, obese patients with breast cancer, colorectal cancer, and endometrial cancer exhibit poorer prognosis [30–32].

This preliminary study was carried out to evaluate the relationship between leptin and its receptor in the progression of ccRCC in a small Malaysian cohort of normal weight and obese ccRCC patients. The findings from this cohort showed that leptin and leptin receptor were not associated with cancer progression in ccRCC tissue. The overall and nuclear expressions of leptin and leptin receptor were not significantly different in ccRCC compared to the adjacent non-cancerous kidney tissue. In addition, overall and nuclear expressions were not significantly associated with pathological stage and grade or BMI for both leptin and leptin receptor. Therefore, the results obtained from this IHC study indicated that leptin and leptin receptor might not be valuable biomarkers for disease progression of ccRCC. The tissue expressions of leptin and its receptor were not predictive of the DSS or RFS among ccRCC patients, but interestingly, high nuclear expression of leptin was associated with worse OS. The contributing factors leading to the association with adverse OS are still unclear, and further studies may be required to confirm this association.

Studies on leptin and leptin receptor, in RCC, have also been performed using other modalities such as serology studies and molecular techniques investigating gene expression. In a gene polymorphism study by Azza et al., in a cohort of 123 RCC and 50 control patients, leptin receptor was considered a potential risk factor for a bad prognosis such as advanced tumour stage, higher nuclear grade, and shorter survival [33]. The results of another gene study carried out specifically in ccRCC by Hui-Jun et al. (77 ccRCC; 161 healthy controls) suggested that the combination of polymorphisms of Lys109Arg and Gln223Arg in leptin receptor can act as a diagnostic and prognostic factor for ccRCC [34].

Serum leptin studies have been carried out in the evaluation of early detection of RCC. Akio et al. evaluated leptin and leptin receptor in the serum of 57 RCC patients. They concluded that both these proteins could have a key role in the invasion of RCC and could be a valuable predictor of prognosis [35]. In comparison, other researchers believed that leptin and its receptor were not valuable biomarkers in the development of this fatal disease. A study by Horiguchi et al. determined that serum leptin was not a useful biomarker for RCC in both males and females [35]. Another researcher found that leptin levels had an inverse association with the risk of RCC [14]. Thus, there are quite disparate outcomes of studies, and this was what prompted our investigation using the IHC technique.

The uniqueness of our current study is that it focused on IHC expression of leptin and its receptor specifically in ccRCC, and a comparison of the protein expressions was carried out between normal and obese patients. We believe that there is valuable information hidden in tissue samples that may contribute tremendously to personalised or precision medicine.

## 5. Conclusion

In conclusion, there were no differential expression patterns of leptin and leptin receptor in ccRCC tissue and adjacent non-cancerous kidney, between non-obese and obese patients. There was also no association with tumour characteristics of ccRCC patients. As a result, both proteins may not have a significant contribution in the formation or progression of ccRCC. However, nuclear expression of leptin was significantly associated with OS in these patients. This may warrant further investigation to elucidate the exact contributing factor for this. There may be involvement of other adipokines secreted from the fat tissue in tumour progression of ccRCC, and further studies should be carried out on those adipokines with a larger patient cohort to identify novel biomarkers for RCC.

## Figures and Tables

**Table 1:** Demographic data of study cohort.

Variable	Subjects (*n* = 60)
Mean age (range) years	60.5 (30-82)
Gender (%)	
Male	42 (70.0)
Female	18 (30.0)
Ethnicity (%)	
Malay	19 (31.7)
Chinese	29 (48.3)
Indian	12 (20.0)
Tumour side (%)	
Right	27 (45.0)
Left	33 (55.0)
BMI (%)	
Normal	26 (41.2)
Obese	34 (56.7)
Mean BMI (range)	27.1 (16.2-40.9)
T stage (%)	
1	29 (48.3)
2	13 (21.7)
3	17 (28.3)
4	1 (1.7)
N stage (%)	
N0	52 (86.7)
N1	8 (13.3)
Metastases (%)	
M0	48 (80.0)
M1	12 (20.0)
Clinical stage (%)	
Stage I	23 (38.3)
Stage II	14 (23.3)
Stage III	12 (20.0)
Stage IV	11 (18.3)
Fuhrman grade (%)	
Grades 1-2	40 (66.7)
Grades 3-4	20 (33.3)

**Table 2:** Expressions of leptin and leptin receptor in tumour tissue with correlation to cohort characteristics and tumour features.

Variables	Leptin						Leptin receptor					
	Overall expression (%) (cytoplasm+membranous)			Nuclear expression (%)			Overall expression (%) (cytoplasm+membranous)			Nuclear expression (%)		
Percentages	Low	High	*p* value	Low	High	*p* value	Low	High	*p* value	Low	High	*p* value
BMI												
Normal	10.0	16.7	0.097	20.0	13.3	0.384	18.3	11.7	0.760	21.7	18.3	0.426
Obese	26.7	15.0		25.0	28.3		21.7	16.7		20.0	26.7	
T stage												
1	20.0	16.7	0.342	18.3	25.0	0.414	15.0	13.3	0.212	21.7	18.3	0.414
2	11.7	3.3		15.0	5.0		8.3	8.3		8.3	11.7	
3	5.0	11.7		10.0	11.7		15.0	6.7		11.7	13.3	
4	0.0	0.0		1.7	0.0		1.7	0.0		0.0	1.7	
N stage												
N0	33.3	26.7	0.523	41.7	33.3	0.084	38.3	25.0	0.374	36.7	36.7	0.483
N1	3.3	5.0		3.3	8.3		1.7	3.3		5.0	8.3	
Metastasis												
M0	30.0	28.3	0.587	33.3	33.3	0.582	28.3	20.0	0.814	35.0	36.7	0.836
M1	6.7	3.3		11.7	8.3		11.7	8.3		6.7	8.3	
Clinical stage												
I	18.3	11.7	0.242	16.7	18.3	0.683	13.3	6.7	0.859	20.0	13.3	0.420
II	11.7	6.7		15.0	5.0		8.3	10.0		8.3	13.3	
III	3.3	10.0		5.0	8.3		10.0	3.3		6.7	11.7	
IV	3.3	3.3		8.3	10.0		8.3	8.3		6.7	6.7	
Grade												
Grades 1-2	28.3	18.3	0.188	28.3	30.0	0.496	26.7	18.3	0.912	26.7	28.3	0.922
Grades 3-4	8.3	13.3		16.7	11.7		13.3	10.0		15.0	16.7	

## Data Availability

The data that support the findings of this study are available on request from the corresponding author. The data are not publicly available due to ethical reason in compromise the privacy of study participants.
